# A comparative analysis of inhibitors of the glycolysis pathway in breast and ovarian cancer cell line models

**DOI:** 10.18632/oncotarget.4499

**Published:** 2015-07-16

**Authors:** Chrysi Xintaropoulou, Carol Ward, Alan Wise, Hugh Marston, Arran Turnbull, Simon P. Langdon

**Affiliations:** ^1^ Division of Pathology, Institute of Genetics and Molecular Medicine, University of Edinburgh, Western General Hospital, Edinburgh, EH4 2XU, UK; ^2^ IOMET Pharma, Nine, Edinburgh BioQuarter, Edinburgh, EH16 4UX, UK; ^3^ Breakthrough Breast Unit, Institute of Genetics and Molecular Medicine, University of Edinburgh, Western General Hospital, Edinburgh, EH4 2XU, UK; ^4^ Current Address: Eli Lilly Research and Development, Windlesham, Surrey, GU20 6PH, UK

**Keywords:** glycolysis, inhibitors, ovarian cancer, breast cancer

## Abstract

Many cancer cells rely on aerobic glycolysis for energy production and targeting of this pathway is a potential strategy to inhibit cancer cell growth. In this study, inhibition of five glycolysis pathway molecules (GLUT1, HKII, PFKFB3, PDHK1 and LDH) using 9 inhibitors (Phloretin, Quercetin, STF31, WZB117, 3PO, 3-bromopyruvate, Dichloroacetate, Oxamic acid, NHI-1) was investigated in panels of breast and ovarian cancer cell line models. All compounds tested blocked glycolysis as indicated by increased extracellular glucose and decreased lactate production and also increased apoptosis. Sensitivity to several inhibitors correlated with the proliferation rate of the cell lines. Seven compounds had IC_50_ values that were associated with each other consistent with a shared mechanism of action. A synergistic interaction was revealed between STF31 and Oxamic acid when combined with the antidiabetic drug metformin. Sensitivity to glycolysis inhibition was also examined under a range of O_2_ levels (21% O_2_, 7% O_2_, 2% O_2_ and 0.5% O_2_) and greater resistance to the inhibitors was found at low oxygen conditions (7% O_2_, 2% O_2_ and 0.5% O_2_) relative to 21% O_2_ conditions. These results indicate growth of breast and ovarian cancer cell lines is dependent on all the targets examined in the glycolytic pathway with increased sensitivity to the inhibitors under normoxic conditions.

## INTRODUCTION

In the 1920s, Otto Warburg demonstrated that cancer cells exhibit an alteration in their metabolism when compared with non-malignant cells. Normal cells in the presence of oxygen use primarily the mitochondrial tricarboxylic acid (TCA) cycle and oxidative phosphorylation for the production of energy and rely on glycolysis only when their oxygen supply is limited. In contrast, cancer cells frequently utilise glycolysis even in the presence of sufficient amounts of oxygen [1, 2]. This persistence of aerobic glycolysis in many cancers is now well substantiated and considered a ‘hallmark’ of advanced cancers [3]. The fact that cancer cells reduce their dependence on mitochondrial oxidative phosphorylation and are more reliant on glycolysis provides a wide range of potential targets for therapy. Targeting aerobic glycolysis is a promising strategy to preferentially kill cancer cells which are dependent on this pathway and in recent years multiple glycolytic inhibitors have been developed [4–6]. However, to date only a few agents have been assessed within *in vivo* experiments and even fewer have undergone clinical trials [4–6].

**Figure 1:**
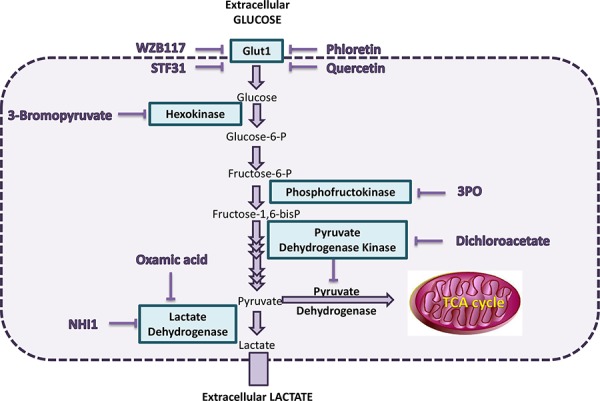
Scheme of selected components of the glycolysis pathway and the inhibitors studied One molecule of glucose is catabolised to two molecules of pyruvate releasing two ATP molecules. Glucose is transported into the cell by the glucose transporter (GLUT) family of molecules and, once inside the cell, is phosphorylated to glucose-6-phosphate (G6P) by hexokinase. After conversion of G6P to fructose-6-phosphate, a second phosphate group is added to fructose-6-phosphate by 6-phosphofructo-1-kinase (PFK1). Several more enzyme reactions eventually produce pyruvate. In normal tissues under aerobic conditions, pyruvate is directed into the mitochondrion by pyruvate dehydrogenase (PDH). Under anaerobic conditions, pyruvate is converted to lactate by lactate dehydrogenase (LDH) and this is also facilitated by the increased activity of pyruvate dehydrogenase kinase 1 (PDHK1), which blocks PDH activity. In malignant cells, this conversion of pyruvate to lactate occurs even under aerobic conditions and has been denoted the Warburg effect.

The glycolytic pathway comprises a series of ten reactions (Figure 1). All of the enzymes within the glycolysis pathway potentially represent targets for anticancer treatment and inhibitors have been developed that target molecular components of this pathway [4–6] (Figure 1). Inhibitors of glucose transporter 1 (GLUT1) include the flavonoids Phloretin and Quercetin [7]. Flavonoids are polyphenolic substances, abundantly distributed in plants, fruits and vegetables and are well known for their powerful anti-oxidative and anti-inflammatory effects [8]. Furthermore, they have been shown to inhibit glucose transmembrane transport and proven to have preclinical anticancer activity [7, 8]. Phloretin, mainly found in the members of the *Rosaceae* family, has been demonstrated to induce apoptosis in breast cancer cells as well as in hepatocellular carcinoma both *in vitro* and *in vivo* [9, 10]. Quercetin has been shown to induce apoptosis in breast and colon cancer cell lines [11, 12]. Recently, Chan *et al*. identified STF31, a compound which induces cell death selectively in VHL-deficient renal cell carcinoma cells by binding specifically to GLUT1 and impairing glucose uptake [13]. WZB117 is a novel specific GLUT1 inhibitor causing suppression of glucose metabolism, inhibition of cellular proliferation both *in vitro* and *in vivo* and cell-cycle arrest leading to senescence and necrosis [14].

Inhibitors of hexokinase II include 3-bromopyruvate (3BP) [15, 16]. This compound has demonstrated anticancer effects both *in vitro* and *in vivo*. It directly inhibits mitochondrial bound hexokinase-II which is up-regulated in many types of cancer. A cytotoxic inhibitory effect has been documented in many types of cancer including melanoma, glioblastoma, mesothelioma, as well as pancreatic, hepatocellular and breast carcinoma [15, 16]. 3-(3-Pyridinyl)-1-(4-pyridinyl)-2-propen-1-one] (3PO) is a novel small molecule inhibitor of PFKFB3, an isozyme which is a component of fructose-2, 6-bisphosphate (Fru-2, 6-BP), an allosteric activator of PFK1 [17]. It has been shown that 3PO suppresses phosphofructokinase activity, decreases glucose uptake and induces growth inhibition in several cell lines, including Ras–transformed cells and additionally in established tumors *in vivo* [17]. PFK158, an optimised 3PO compound, is now undergoing a clinical trial [18].

Dichloroacetate (DCA) is a pyruvate analogue which inhibits pyruvate dehydrogenase kinase (PDHK1), an enzyme which inhibits the mitochondrial pyruvate dehydrogenase (PDH). In this way it suppresses glycolysis and stimulates oxidative phosphorylation. It is reported to have antitumor activity both *in vitro*, in several types of cancer, and *in vivo* [19, 20]. DCA is now currently undergoing clinical trials [21] and while promising results were obtained in 3 of 5 glioblastoma patients treated with DCA alongside temozolomide and radiotherapy [22], combination therapy trials with platinum have so far failed to show activity against non-small cell lung cancer [23]. Furthermore, the drug is not without toxicity and at high concentrations produces peripheral neuropathy [22]. Oxamic acid is an established pyruvate analogue and a competitive lactate dehydrogenase (LDH) inhibitor. Some promising anti-proliferative effects have been documented *in vitro* using cervical adenocarcinoma and hepatocellular carcinoma cell lines [24, 25]. In 2011 Granchi *et al*. published the identification of several novel N-hydroxyindole-based (NHI) LDH Inhibitors [26]. NHI-1 (aka compound 1j) is a competitive isoform selective LDH A inhibitor [26]. It has been shown to diminish lactate production and inhibit cell proliferation in a variety of cancer cell lines [26, 27].

This study examined the effects of these inhibitors on breast and ovarian cancer cell lines by using cell proliferation, glucose and lactate assays, to assess impact on aerobic glycolysis. While there are reports of these agents showing activity against various cancer cell line models, we are not aware of any detailed comparative studies of multiple inhibitors against panels of either breast or ovarian cancer cell line models. We sought to understand why certain cell lines were more sensitive to inhibitors of these pathways.

Metformin is a biguanide widely prescribed for the treatment of type 2 diabetes mellitus. An increasing number of epidemiologic studies have associated metformin with a decreased risk of several types of cancer and improved clinical outcome in diabetic cancer patients [28]. Metformin inhibits the mitochondrial respiratory chain complex I and its anticancer effect is mainly attributed to the activation of the AMP- activated protein kinase (AMPK) resulting in decreased hepatic gluconeogenesis [28–30]. Here we hypothesized that since metformin decreases ATP production, inhibiting mitochondrial oxidative phosphorylation, a combined inhibition of the glycolytic pathway could possibly result in a complete depletion of cellular ATP and lead to increased cell death.

An additional objective of this study was to evaluate the effect of these inhibitors under varying oxygenation levels. Previous reports have revealed considerable intratumoral heterogeneity in oxygenation status, as well as increased variability among individual solid tumors [31]. For breast cancers, Vaupel *et al*. reported marked variability in oxygen levels (from 13%–0% oxygen) even when comparing tumors of the same stage, grade and histology. Furthermore, they demonstrated great heterogeneity in the oxygenation of individual tumors with hypoxic and anoxic areas distributed within the same tumor tissue [31]. Hypoxia is a well substantiated common feature of the tumor microenvironment that contributes to resistance to radiotherapy, chemotherapy and tumor relapse. The transcription factor HIF-1 (Hypoxia Inducible Factor 1) is induced by hypoxia and regulates expression of proteins that enable cells to survive hypoxia [32]. Many of the glycolytic enzymes, including all the targets selected here (GLUT1, Hexokinase II, PFKFB3, PDHK1 and LDHA), are known to be HIF1-inducible [32]. Previous studies using these inhibitors have demonstrated both reduced and increased potency in hypoxic conditions dependent on the inhibitor and cancer model, hence we were interested to ascertain their effects in breast and ovarian cancer cell line models. To date, most experimental studies have been carried out under normoxic conditions. This study investigated the sensitivity of breast cancer cells to glycolytic inhibitors at a range of oxygen levels (21% −7% −2% −0.5% O_2_), the latter three representing more closely the microenvironment of clinical cancers.

## RESULTS

### Targeted inhibition of the glycolysis pathway in breast and ovarian cancer cell lines

**Figure 2:**
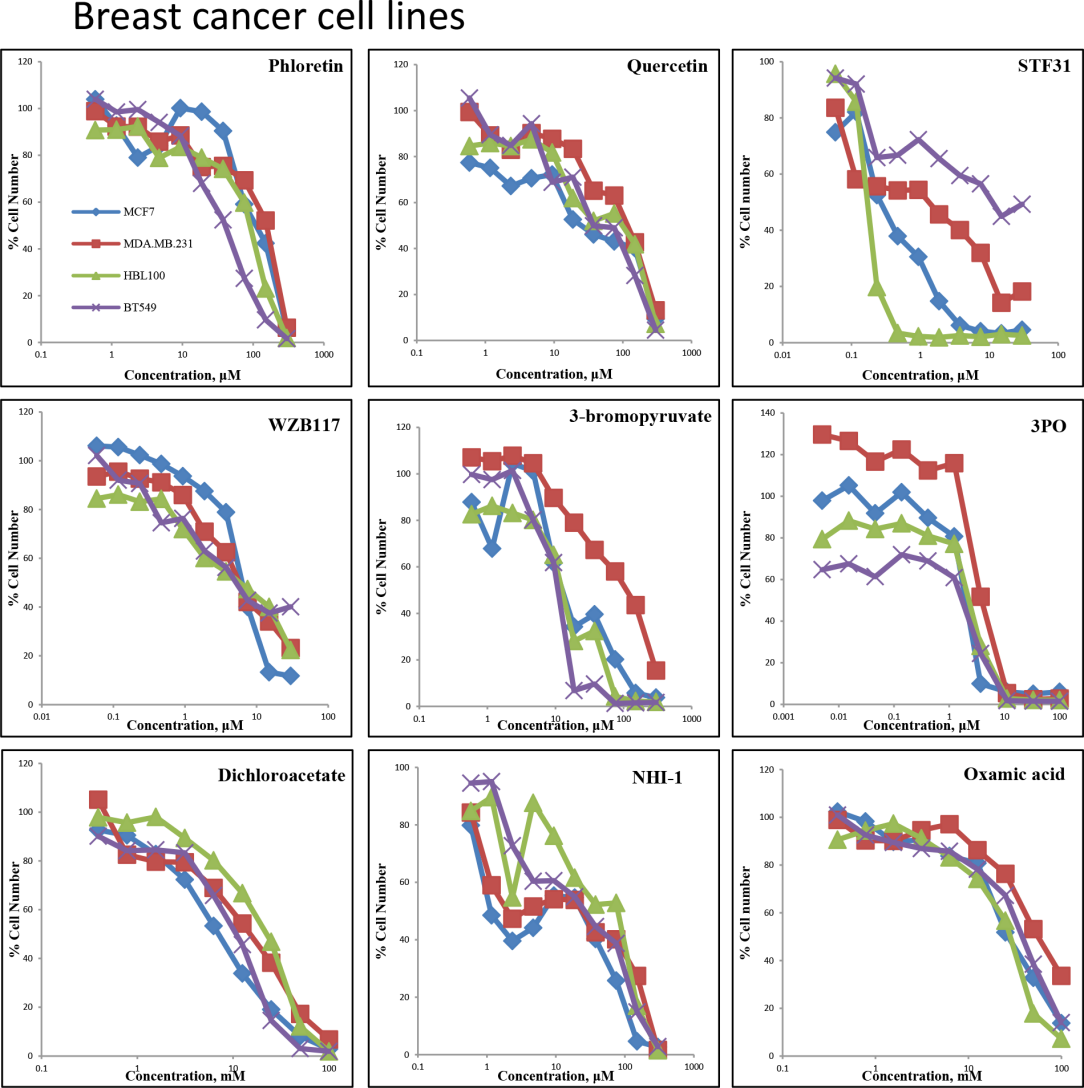
Concentration response curves of four breast cancer cell lines (MCF-7, MDA-MB-231, HBL100, BT549) treated with nine glycolytic inhibitors Breast cancer cells were treated with Phloretin concentrations between 0.6–300 μM, Quercetin concentrations between 0.6–300 μM, STF31 concentrations between 0.06–30 μM, WZB117 concentrations between 0.06–30 μM, 3BP concentrations between 0.6–300 μM, 3PO concentrations between 0.005–100 μM, DCA concentrations between 0.4–100 mM, Oxamic acid concentrations between 0.2–100 mM and NHI-1 concentrations between 0.6–300 μM. An SRB assay was performed on day 5. Results shown here are in replicates of 6. Constant 0.3% DMSO concentration was used across the whole curve for Phloretin, Quercetin, STF31 and NH-1. Constant 0.3% ethanol concentration was used across the whole curve for WZB117.

**Figure 3:**
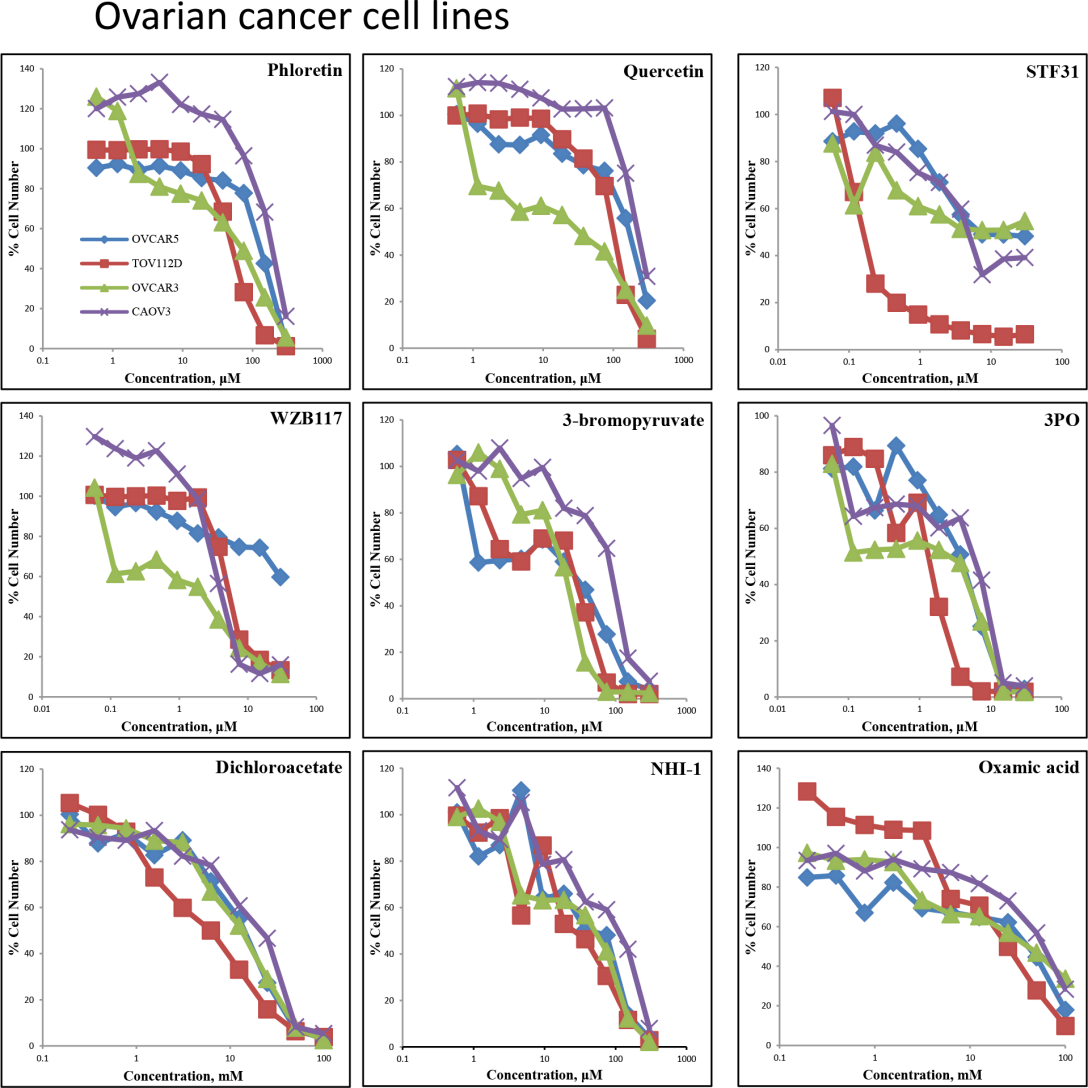
Concentration response curves of four ovarian cancer cell lines (OVCAR5, TOV112D, OVCAR3, CAOV3) treated with nine glycolytic inhibitors Ovarian cancer cells were treated with Phloretin concentrations between 0.6–300 μM, Quercetin concentrations between 0.6–300 μM, STF31 concentrations between 0.06–30 μM, WZB117 concentrations between 0.06–30 μM, 3BP concentrations between 0.6–300 μM, 3PO concentrations between 0.06–30 μM, DCA concentrations between 0.4–100 mM, Oxamic acid concentrations between 0.2–100 mM and NHI-1 concentrations between 0.6–300 μM.An SRB assay was performed on day 5. Results shown here are in replicates of 6. Constant 0.3% DMSO concentration was used across the whole curve for Phloretin, Quercetin, STF31 and NH-1. Constant 0.3% ethanol concentration was used across the whole curve for WZB117

The effect of inhibitors targeted against upstream components of the glycolysis pathway (GLUT1, hexokinase II, PFKFB3) and the downstream component LDH-A were compared on cell proliferation. These were also compared with an inhibitor (DCA) of PDHK1 which promotes conversion of pyruvate to acetyl-CoA. Proliferation was assessed by use of a sulphorhodamine B (SRB) assay and growth was assayed after a 5-day treatment period. Concentration response curves for the breast and ovarian cancer cell lines (Supplementary Table 1) are illustrated in Figures 2 and 3 respectively and IC_50_ values are recorded in Table 1.

**Table 1:** Summary of the IC_50_ concentrations obtained from four breast and four ovarian cancer cell lines when treated with the indicated glycolytic inhibitors for 5 days

	MCF-7	MDA-MB-231	HBL100	BT549	OVCAR5	TOV112D	OVCAR3	CAOV3
Phloretin (μM)	122	135	69	36	119	51	54	197
Quercetin (μM)	44	105	76	44	154	94	21	240
STF31 (μM)	0.31	0.81	0.17	18	7.4	0.15	>30	4.9
WZB117 (μM)	6.4	6.3	5.2	6.6	>30	5.9	1.4	4.3
Bromopyruvate (μM)	18	84	15	10	20	16	20	84
3PO (μM)	2.1	3.8	2.7	2.3	3.9	1.2	2.8	5.8
Dichloroacetate (mM)	6.8	13	19	9.1	14	5.5	13	20
Oxamic acid (mM)	28	58	27	39	38	24	34	59
NHI-1 (μM)	8	20	53	22	45	28	35	91

Four breast cancer cell lines (MCF-7, MDA-MB-231, HBL100 and BT549) were first investigated (Figure 2). Four GLUT1 inhibitors, Phloretin, Quercetin, STF31 and WZB117, were compared. The flavonoids Phloretin and Quercetin had similar effects on the growth of the cell lines. For Phloretin, the IC_50_ values ranged between 36–135 μM while IC_50_ values ranged between 44–106 μM for Quercetin. STF31 produced a much larger differential effect between the cell lines. MCF-7 and HBL100 cells were very sensitive while BT549 cells showed resistance to the compound. The IC_50_ values varied markedly and ranged between 0.2–18 μM. In contrast, WZB117 had a very similar effect on all four cell lines with IC_50_ values ranging between 5.2–6.6 μM. 3BP which targets hexokinase II had IC_50_ values that ranged between 10–84 μM; MCF-7, HBL100 and BT549 cells had a similar response, while MDA-MB-231 cells were more resistant. All cell lines responded in a similar way to 3PO which targets PFKFB3 having IC_50_ values between 2.1–3.8 μM. For DCA the concentrations needed were higher compared to the previous inhibitors and were in the millimolar concentration range. MCF-7 proved the most sensitive breast cancer cell line (IC_50_ 6.8 mM), while HBL100 (IC_50_ 18.9 mM) was least sensitive. Finally, for NHI-1 and Oxamic acid which target LDH-A, the IC_50_ values ranged between 27–58 mM for Oxamic acid and between 8–53 μM for NHI-1. The MDA-MB-231 cell line was found more resistant to several of the inhibitors.

For the 4 ovarian cancer cell lines, similar datasets were obtained (Figure 3). Phloretin inhibited cell proliferation with IC_50_ values ranging between 51–197 μM and Quercetin with values between 21–240 μM. STF31 and WZB117 produced increased differential effects between the cell lines. The IC_50_ values ranged between 0.2 to greater than 30 μM for STF31 and between 1.4 to greater than 30 μM for WZB117. OVCAR3 cells demonstrated resistance to STF31 and OVCAR5 cells were resistant to WZB117. For 3BP, the IC_50_ values ranged between 16–84 μM with the CAOV3 cell line demonstrating most resistance to this compound. Regarding 3PO, the TOV112D cell line was the most sensitive with IC_50_ value almost 1.2 μM while CAOV3 was least sensitive, with an IC_50_ 5 times higher. DCA and Oxamic acid again required much greater (millimolar) concentrations compared to the other compounds. In both cases, TOV112D cell line showed the lowest IC_50_ values (5.5 mM for DCA and 24 mM for Oxamic acid) while CAOV3 had the highest (20 mM for DCA and 59 mM for Oxamic acid). Finally, for NHI-1 the IC_50_ values ranged between 28–91 μM.

### Glucose uptake and lactate production are inhibited by all of the studied inhibitors

**Figure 4:**
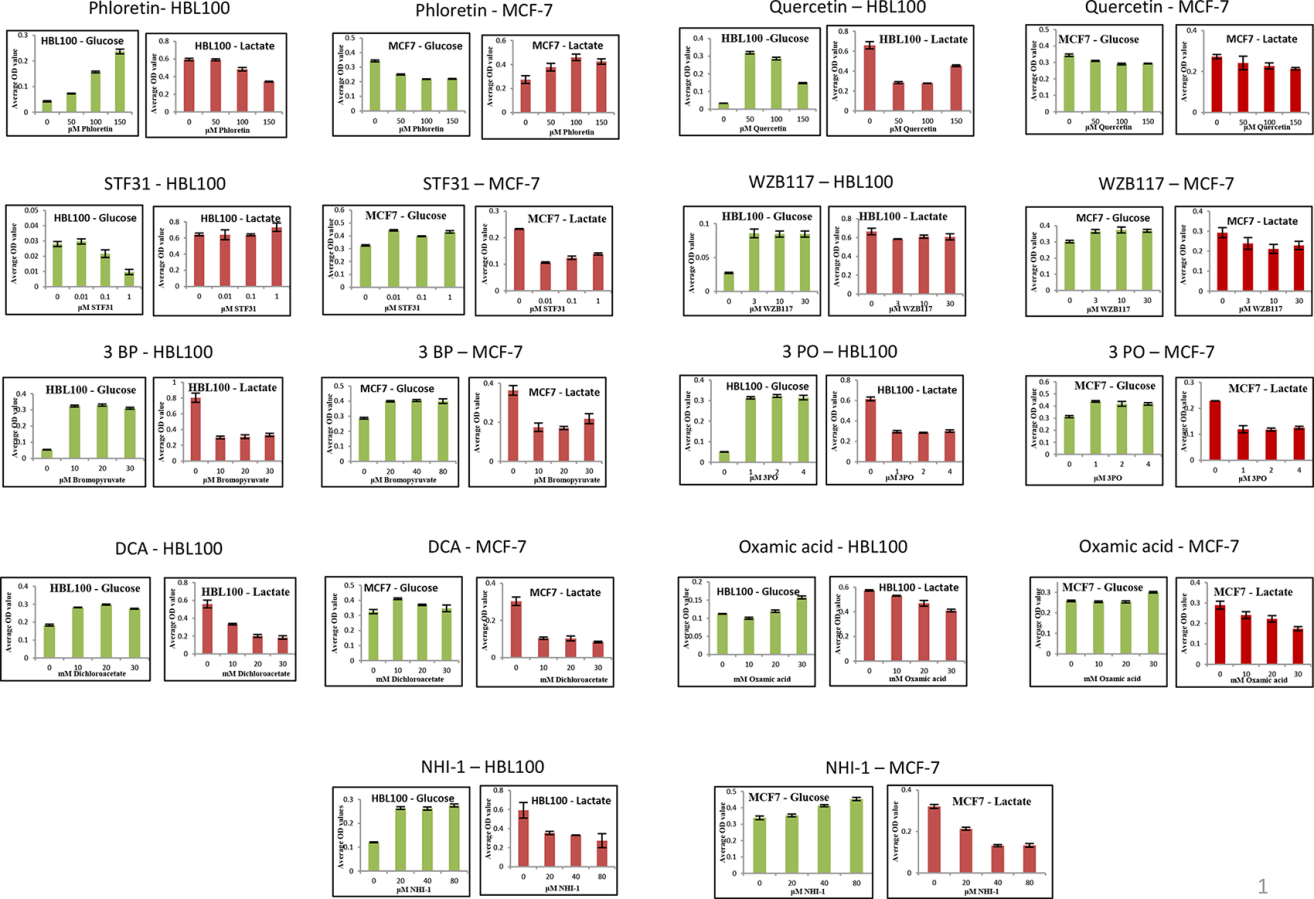
Extracellular glucose remaining in the media and extracellular lactate secreted against increasing concentration of the nine glycolytic inhibitors after a 24 h treatment of MCF-7 or HBL100 cells Results are reported as the mean of three repeats and error bars represent standard deviations.

The effects of the above inhibitors were next examined on glucose uptake and lactate production in HBL100 and MCF-7 cells (Figure 4). Glucose concentrations remaining in the media and extracellular lactate production into the medium after a 24 h treatment with the indicated compound are shown. The range of concentrations used for each compound was based on the corresponding IC_50_ values, derived from the SRB assay, for each specific cell line. Both sets of measurements were conducted on the same day from the same sample.

All the inhibitors tested proved effective in blocking glycolysis in at least one of the two cell lines tested (Figure 4). Increasing concentrations of Phloretin caused an increase in the glucose remaining in the media while lactate production decreased following the same pattern. Regarding the other three glucose transport inhibitors Quercetin, STF31 and WZB117, even the lowest concentration used produced a maximal effect causing accumulation of glucose and depletion of lactate in the culture media. 3BP, 3PO and DCA were also effective in inhibiting glycolysis. Low concentrations of each compound caused a large increase in the glucose accumulating in the media and a corresponding decline in the secreted lactate. The inhibition of glycolysis hit a plateau with treatment of 10 μM 3BP, 10 μM 3PO or 10 mM DCA and no apparent additional effect was detected with increasing concentrations. The lactate dehydrogenase inhibitors likewise suppressed glycolysis. Following treatment with NHI-1 and Oxamic acid, cells demonstrated a modest increase in glucose and a more profound depletion of lactate in their culture media.

### Glycolytic inhibitors induced apoptosis

**Figure 5:**
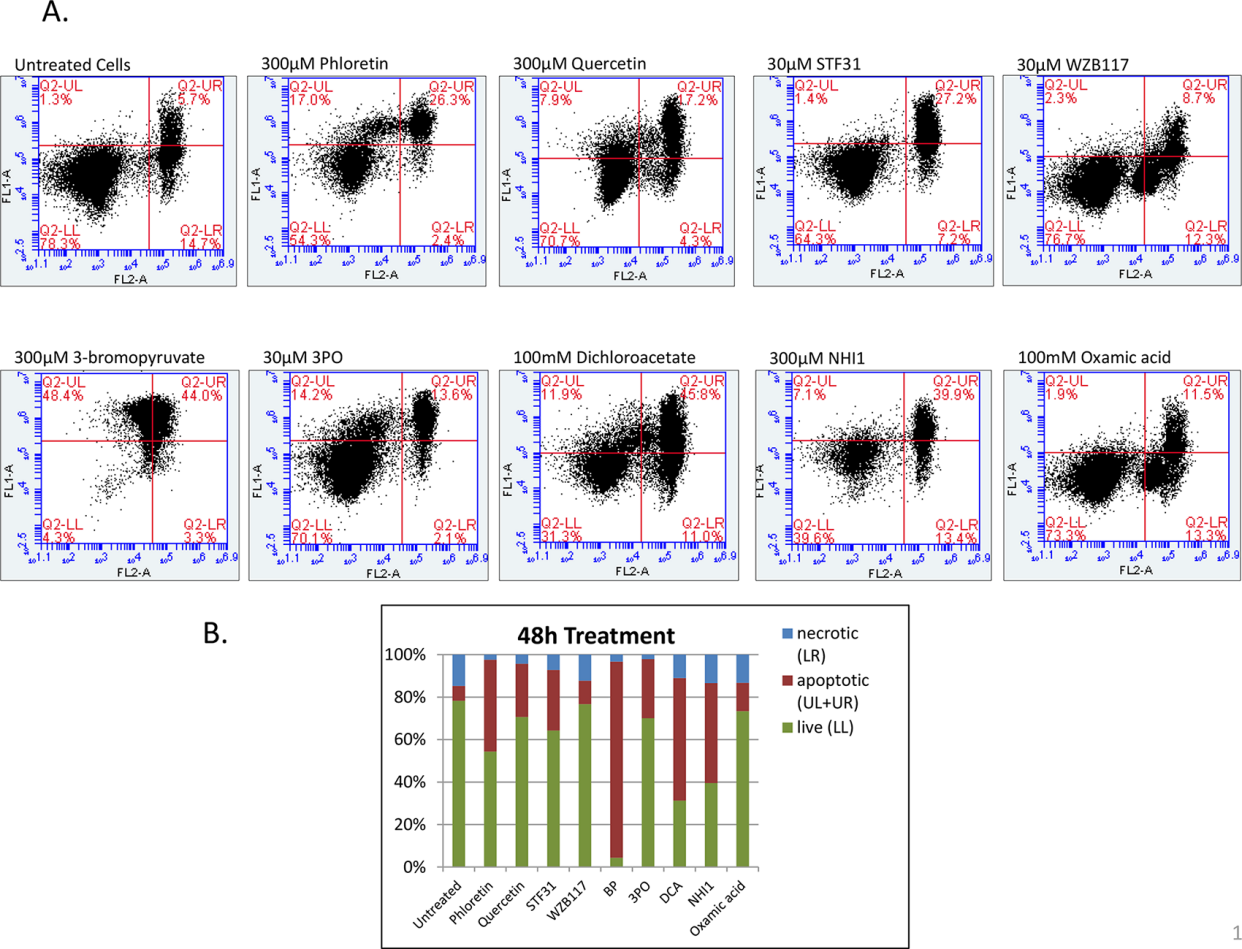
A. Two- dimensional scatter plots of Annexin V (FL1) vs PI (FL2) generated from flow cytometry analysis of MCF-7 cells when treated with 9 glycolytic inhibitors for 48 h After treatment cells were harvested, washed with cold PBS and resuspended in 1X Binding Buffer. 5 μl of FITC Annexin and 5 μl of PI were added to the suspension and cells were incubated for 15min in the dark prior to analysis with the BD Accuri C6. The numbers in the quadrants of each plot indicate the percentage of cells in this region. Cells in LL represent intact viable cells (Annexin negative and PI negative), in UL represent early apoptotic cells (Annexin positive and PI negative), in UR represent late apoptotic cells (Annexin positive and PI positive) and in LL represent necrotic cells (Annexin negative and PI positive). **B.** Percentage of apoptotic, necrotic and live cells after a 48 h treatment with 300 μM Phloretin, 300 μM Quercetin, 30 μM STF31, 30 μM WZB117, 300 μM 3BP, 30 μm 3PO, 100 mM DCA, 300 μM NHI-1 and 100mM Oxamic acid compared to untreated cells.

To investigate whether the growth inhibitory effect of these compounds was associated with induction of apoptotic cell death, flow cytometric analysis was performed. MCF-7 cells were treated with 9 glycolytic inhibitors for 48 h, stained with FITC-conjugated Annexin V and PI and analysed using flow cytometry. Cells were separated into four different groups, the lower left quadrant represents intact viable cells (Annexin negative and PI negative), the upper left quadrant represents early apoptotic cells (Annexin positive and PI negative), the upper right quadrant represents late apoptotic cells (Annexin positive and PI positive) and the lower right quadrant represents necrotic cells (Annexin negative and PI positive). Cells stained with Annexin-FITC were collectively considered as apoptotic cells (upper region). As shown in Figure 5A there is an induction of both early and late phases of apoptosis after treatment with the glycolytic inhibitors compared to untreated cells. Untreated cells showed 7% apoptosis (Annexin positive) whereas for cells treated with 300 μM Phloretin the percentage of apoptotic cells increased to 43%, with 300 μM Quercetin to 25%, with 30 μM STF31 to 29%, with 30 μM WZB117 to 11%, with 300 μM 3BP to 92%, with 30 μM 3PO to 28%, with 100mM DCA to 58%, with 300 μM NHI-1 to 47% and with 100mM Oxamic acid to 13% (Figure 5B).

### Correlation analysis of IC_50_ concentrations and cell proliferation rate

**Figure 6:**
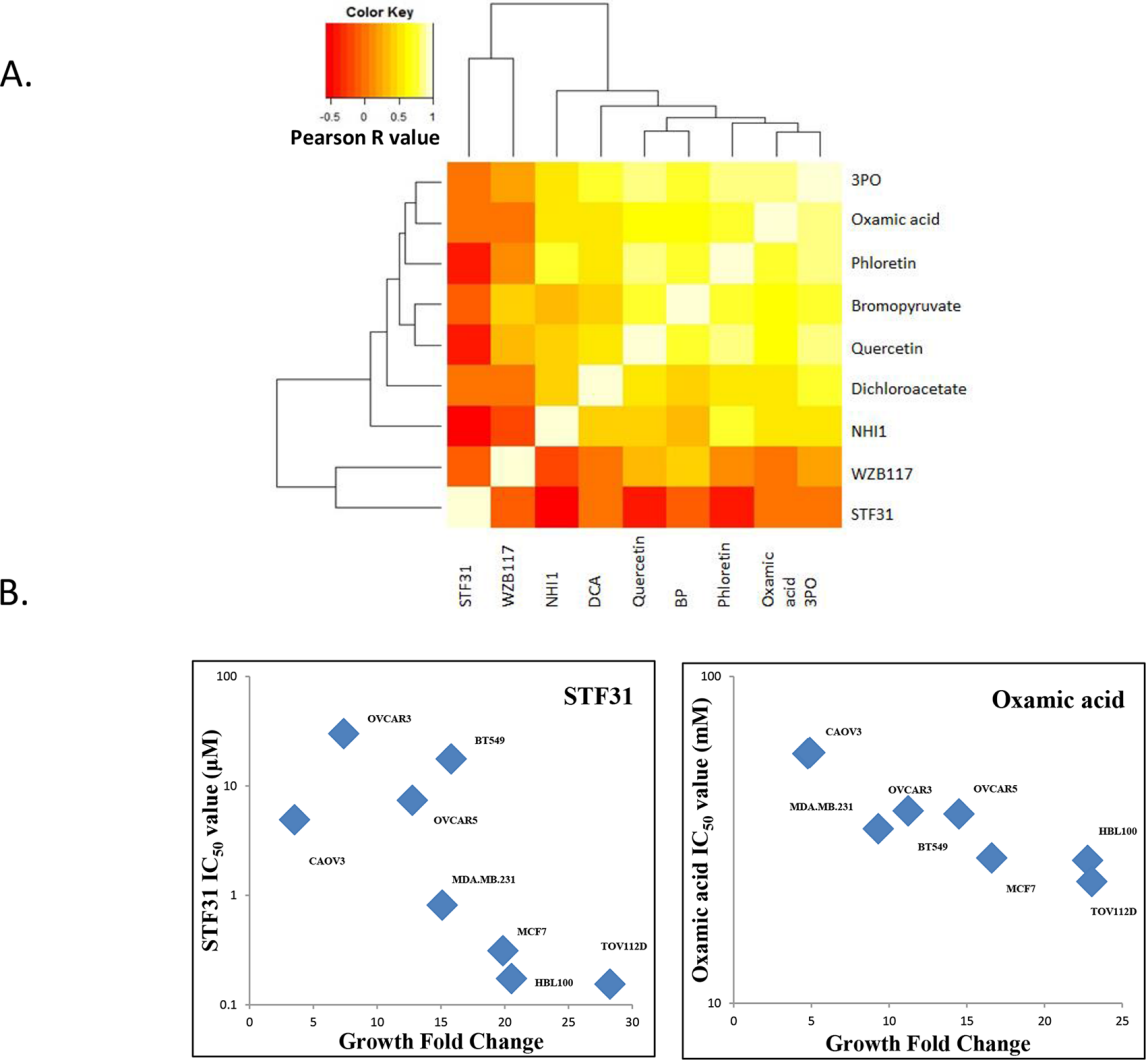
A. Correlation heat-map demonstrating that seven glycolytic inhibitors had IC_50_ concentrations that correlated with each other in the panel of cell lines This is consistent with a shared mechanism of action. Dark orange colours indicate negative Pearson R correlation values while bright white colours indicate positive Pearson R correlation values. **B.** STF31 and Oxamic acid IC_50_ values of the panel of four breast and four ovarian cancer cell lines plotted against the growth fold change of the respective cell line. Non-parametric Spearman correlation *r* = −0.7619 with two-tailed *P* value 0.0368 for STF31; considered significant. Non-parametric Spearman correlation *r* = −0.9048 with two-tailed *P* value 0.0046 for Oxamic acid; considered very significant.

The association of IC_50_ values against the panel of 8 cell lines was assessed for each pair of drugs indicating that sensitivity to 7 of these inhibitors correlated with each other (Supplementary Table 2). The correlation heatmap in Figure 6A illustrated that 7 inhibitors (Phloretin, Quercetin, 3BP, 3PO, DCA, NHI-1 and Oxamic acid) had IC_50_ concentrations that gave high Pearson R correlation values when compared to each other. This is consistent with these agents sharing a common mechanism of action targeting the same pathway. STF31 and WZB117 did not significantly correlate with any of the other agents. The expression levels of GLUT1, hexokinase II, PFKFB3, PDHK1 and LDHA were examined in the cell line panel (Supplementary Figure 1). No significant correlation between the expression of the targets in the eight cell lines and their sensitivity to the inhibitors was detected (data not shown). Sensitivity to STF31 and Oxamic acid was found to correlate significantly with the proliferation rate of the cell lines, giving *p* values of 0.0368 and 0.0046 respectively. The fastest growing cell lines were more sensitive to these compounds while the slowest growing cell lines presented greater resistance (Figure 6B).

### Combination of metformin and glycolytic inhibitors synergistically inhibited cancer cell growth of a triple negative breast cancer cell line

**Figure 7:**
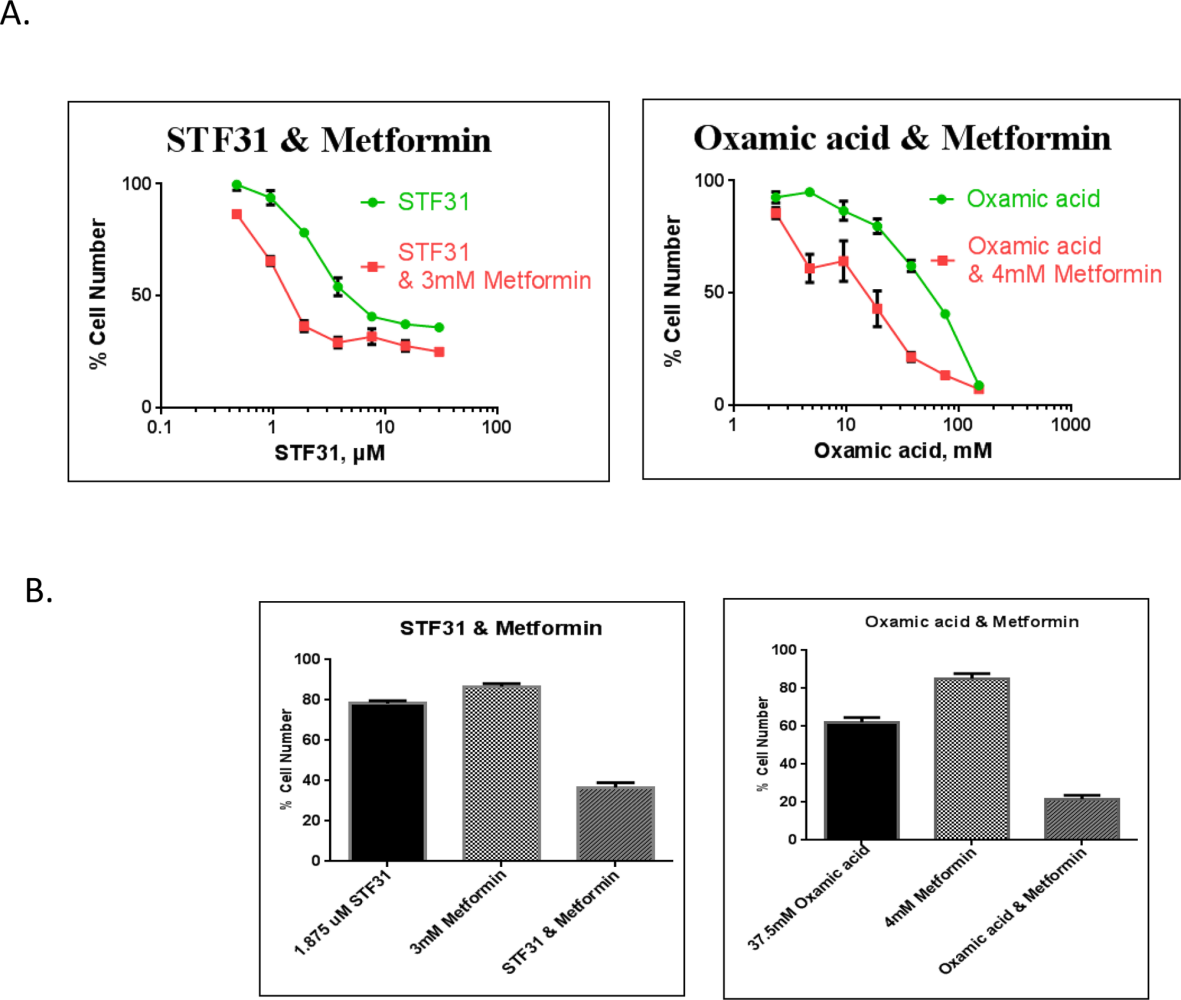
A. Concentration response curves of drug interaction studies MDA-MB-231 cells were treated with STF31 concentrations between 0.5–30 μM and Oxamic acid concentrations between 2.3–150 mM alone or in combination with a constant concentration of 3mM or 4 mM metformin respectively. An SRB assay was performed on day 3. Error bars shown here represent SD (*n* = 6). **B.** Effect of 2 μM STF31 alone or in combination with 3 mM metformin and of 38 mM Oxamic acid alone or in combination with 4 mM metformin on cell viability. Both combinations are characterised synergistic based on their CI values generated using the Calcusyn Software. Error bars shown here represent SD (*n* = 6).

The interaction between glycolytic inhibitors and the antidiabetic drug metformin was examined. A range of different concentrations of two glycolytic inhibitors, STF31 and Oxamic acid, was used in combination with a constant fixed concentration of metformin and incubation lasted for 72 h. Metformin enhanced the potency of both STF31 and Oxamic acid to inhibit cancer cell proliferation compared to the effect of these drugs individually (Figure 7A). To evaluate the efficacy of the combinations, data were analysed using the Calcusyn Software and Combination Index (CI) values were generated (Tables 2a, 2b). Examples of synergistic combinations are depicted in Figure 7B. For example, 1.9 μM of STF31 alone reduced the percentage of cell number to 78% and 3mM of metformin to 87% while the combination of both drugs reduced the cell number to 37% compared to untreated cells. For this combination a CI value equal to 0.182 is generated which is characterised as strong synergy. Similarly, 37.5 mM of Oxamic acid reduced cell number to 62% and 4 mM of metformin to 85% while their combination reduced the cell number to 22%. The CI value generated for this combination was 0.336 characterised as synergy.

**Table 2:** Summary of drug interaction studies

a.			
STF31 (μM)	Metformin (μM)	Fa	CI
0.46875	3000	0.134506	**1.183**
0.9375	3000	0.343076	**0.36**
1.875	3000	0.63395	**0.182**
3.75	3000	0.706688	**0.251**
7.5	3000	0.68129	**0.522**
15	3000	0.721799	**0.882**
30	3000	0.747518	**1.585**

b.			
Oxamic acid (mM)	Metformin (mM)	Fa	CI
2.34375	4	0.144005	**1.683**
4.6875	4	0.389971	**0.467**
9.375	4	0.356693	**0.73**
18.75	4	0.568973	**0.483**
37.5	4	0.783404	**0.336**
75	4	0.864651	**0.382**
150	4	0.926606	**0.411**

Tables show concentration range of STF31 (2a) and Oxamic acid (2b) used with a constant concentration of Metformin. FA-fraction affected represents the growth inhibition for each tested combination and CI represents the combination index values generated through Calcusyn. Values lower than 0.8 indicate synergism, values between 0.8 and 1.2 indicate additivity and values higher than 1.2 indicate antagonism

### Growth inhibition at varying oxygen tensions

**Figure 8:**
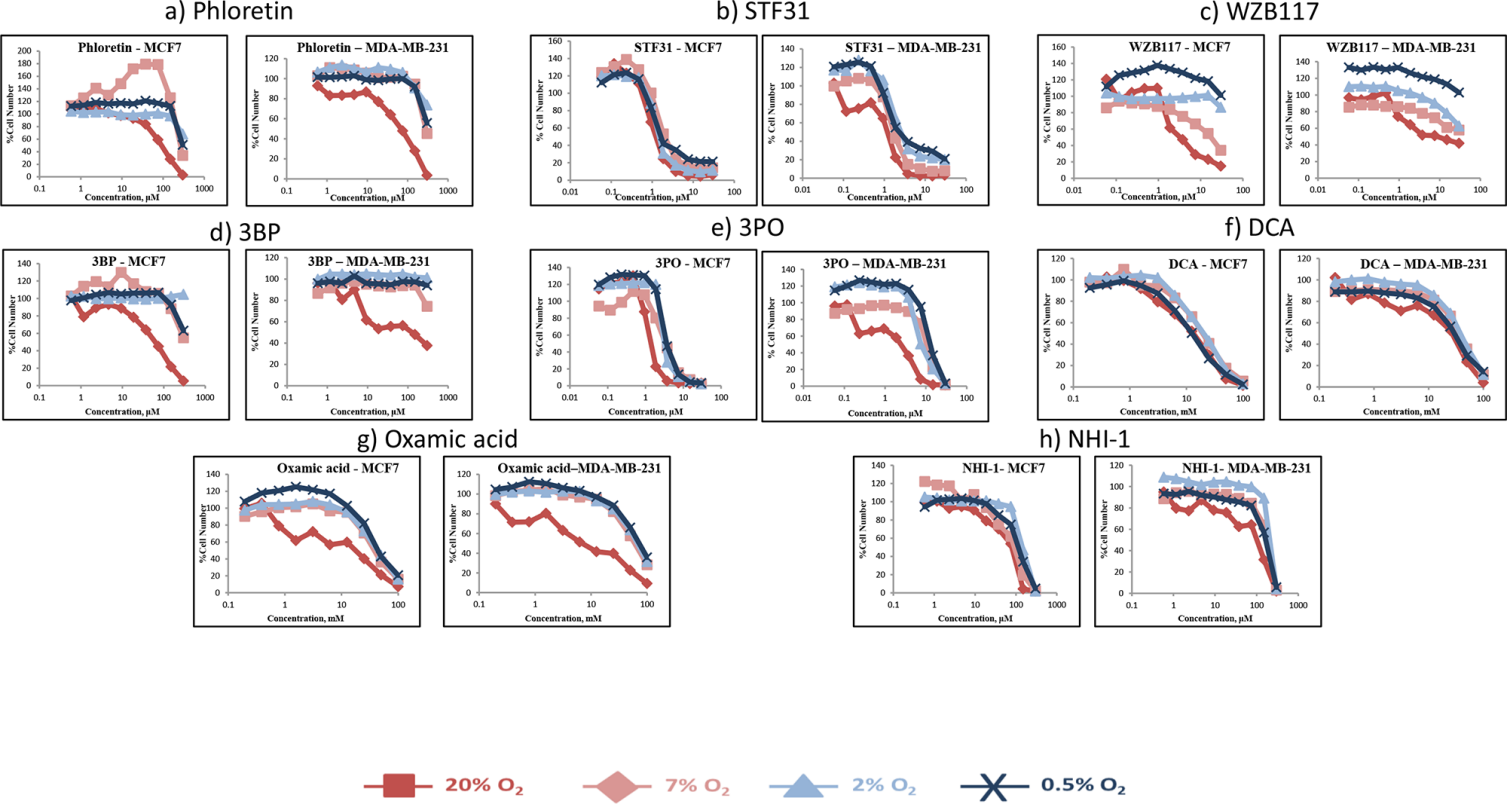
Concentration response curves of two breast cancer cell lines (MCF-7, MDA-MB-231) treated with eight glycolytic inhibitors under four different oxygen conditions (21% O_2_, 7% O_2_, 2% O_2_, 0.5% O_2_) Breast cancer cells were treated with STF31 concentrations between 0.06–30 μM (Figure 8a), WZB117 concentrations between 0.06–30 μM (Figure 8b), Phloretin concentrations between 0.6–300 μM (Figure 8c), 3BP concentrations between 0.6–300 μM (Figure 8d), 3PO concentrations between 0.06–30 μM (Figure 8e), DCA concentrations between 0.2–100 mM (Figure 8f), Oxamic acid concentrations between 0.2–100 mM (Figure 8g) and NHI-1 concentrations between 0.6–300 μM (Figure 8h). An SRB assay was performed on day 5. Results shown here are in replicates of 6. Constant 0.3% DMSO concentration was used across the whole curve in Figures 8a, 8c, 8e and 8h. Constant 0.3% Ethanol concentration was used across the whole curve in Figure 8b.

We next sought to examine the effect of the oxygen level on sensitivity to these glycolytic inhibitors. For this purpose two breast cancer cell lines were selected, the ER positive, luminal A MCF-7 and the basal B, triple negative MDA-MB-231 lines. The breast cancer cells were adapted to the different oxygen conditions, before being treated with eight of the glycolytic inhibitors. The SRB assay was performed after a five day treatment. In Figure 8 is depicted the percentage of cell number for the two cell lines against increasing concentration of each of the eight compounds under four different oxygen conditions 21% O_2_, 7% O_2_, 2% O_2_, 0.5% O_2_. Table 3 presents the corresponding IC_50_ values.

**Table 3:** Summary of the IC_50_ concentrations presented from two breast cancer cell lines (MCF-7, MDA-MB-231) when treated with the indicated glycolytic inhibitors for 5 days under four different oxygen conditions (21% O_2_, 7% O_2_, 2% O_2_, and 0.5% O_2_)

IC_50_	MCF-7				MDA-MB-231			
	21% O_2_	7% O_2_	2% O_2_	0.5% O_2_	21% O_2_	7% O_2_	2% O_2_	0.5% O_2_
**STF31 (μM)**	1.284	2.277	1.445	1.613	1.243	1.803	2.367	2.162
**WZB117 (μM)**	2.3	16.419	>30	>30	4.079	N/A	>30	N/A
**Phloretin (μM)**	90.016	295.224	>300	>300	69.644	289.107	>300	>300
**BP (μM)**	62.659	>300	N/A	>300	38.989	>300	N/A	>300
**3PO (μM)**	1.34	3.759	3.481	3.673	1.486	11.232	8.574	12.9
**DCA (mM)**	13.043	18.91	19.558	12.569	23.3	38.904	36.967	31.079
**NHI1 (μM)**	61.778	80.026	150.111	111.495	93.628	190.059	227.572	174.091
**Oxamic acid(mM)**	10.4	39.368	47.089	49.478	6.944	60.431	66.192	73.993

Both cell lines were sensitive to all the inhibitors in hypoxic conditions. The most striking observation was the increase presented in the IC_50_ values between 21% O_2_ and 7% O_2_. Both cell lines when treated with the eight inhibitors under 7% O_2_ followed the same pattern and their IC_50_ values were increased from 1.5 fold (MDA-MB-231 cells treated with STF31 – Figure 8b) up to 9 fold (MDA-MB-231 cells treated with Oxamic acid – Figure 8g) with an average of 4 fold. (Table 3)

The response of the breast cancer cell lines at the lower oxygen levels (2% O_2_ and 0.5% O_2_) differed between the compounds. In some cases, cells became even more resistant to the compounds increasing further their IC_50_ value (e.g. MDA-MB-231 cells treated with Oxamic acid – Figure 8g), or presented a modest decrease (e.g. MDA-MB-231 cells treated with DCA – Figure 8f). In the majority of the treatments, response between the three lower levels was similar. Both cell lines were more resistant to the whole panel of glycolytic inhibitors under any of the lower oxygen levels tested compared to 21% O_2_ conditions (Table 3).

When drug IC_50_ was correlated with growth rate under the range of differing O_2_ concentrations for both cell lines, there was in general a significant inverse association between higher growth rates and lower IC_50_ with increasing oxygen level (Supplementary Table 3).

### Modulation of target expression levels under different O_2_ levels

We speculated that decreased sensitivity to the glycolytic inhibitors in hypoxic conditions could be attributed to up-regulation of the respective target. In an attempt to understand the increased resistance to the inhibitors under lower levels of oxygen the expression of the five glycolytic targets of interest was examined in these conditions.

**Figure 9:**
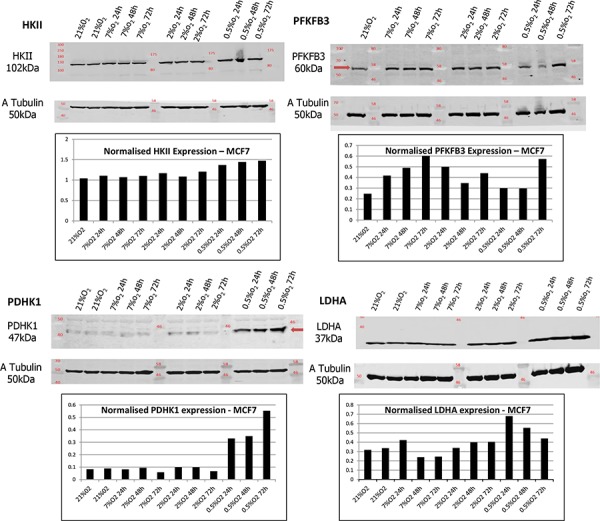
HKII, PFKFB3, PDHK1 and LDHA expression were examined in MCF-7 cells at different O2 levels Lysates were taken from cells cultured in 0.5% O2, 2% O2 and 7% O2 for different periods of time, 24 h, 48 h and 72 h. Samples are presented as follows: 21% O2, 21% O2, 7% O2 24 h, 7% O2 48 h, 7% O2 72 h, 2% O2 24 h, 2% O2 48 h, 2% O2 72 h, 0.5% O2 24 h, 0.5% O2 48 h and 0.5% O2 72 h. Tubulin expression was examined in the same samples as a loading control. Densitometric analysis of HKII, PFKFB3, PDHK1 and LDHA expression was performed using the Odyssey Infrared Imaging System software (Licor).

Expression of the GLUT1 transporter as well as of the Hexokinase II, PFKFB3, PDHK1 and LDHA enzymes were examined in MCF-7 and MDA-MB-231 cells cultured in 0.5% O_2_, 2% O_2_ as well as 7% O_2_ for different periods of time, 24 h, 48 h and 72 h and compared with expression at 21% O_2_ (Figure 9 - Supplementary Figures 2, 3). PFKFB3 and PDHK1 were up-regulated under hypoxic conditions in both cancer cell lines, while Hexokinase II and LDHA were up-regulated only in MCF-7 cells at 0.5% O_2_.

## DISCUSSION

The purpose of this study was to evaluate the effects of a series of inhibitors targeted against multiple points of the glycolysis pathway in breast and ovarian cancer cell line models. All inhibitors induced apoptosis and blocked glycolysis irrespective of their target and point of action in the pathway. The majority of these compounds had IC_50_ concentrations that correlated significantly with each other consistent with sharing a common mechanism of action. A second objective was to examine the effect of selected glycolytic inhibitors in combination with the antidiabetic drug metformin. The third aim of the study was to investigate the impact of varying levels of O_2_ on inhibitor efficacy - increasing concentrations of oxygen resulted in more rapid cell growth rates and increased potency of the inhibitors.

This study provides confirmation that all 9 of these inhibitors block the glycolytic pathway. All the tested inhibitors caused glucose accumulation in the media of cultured cells combined with a decline in the production of lactate, the final product of glycolysis (Figure 4). It is noteworthy that even when targeting the final step of the pathway an effect on the uptake of glucose can be observed. This is attributed to the tight allosteric regulation of the key glycolytic enzymes [33]. All the tested compounds inhibited cell proliferation of both breast and ovarian cancer cells, in a concentration-dependent manner at concentrations associated with glycolytic inhibition (Figures 2, 3). Each of the inhibitors presented a different potential in attenuating cell proliferation. More recently developed compounds (STF31, WZB117, 3PO and NHI-1) proved more potent and inhibited tumor cell growth at lower concentrations. The compounds Phloretin, 3PO, DCA, NHI1 and 3BP had clear cytotoxic effects on the majority of cell lines while Quercetin, WZB117 and Oxamic acid showed cytostatic effects (judged based on the comparison of the cell number on day 0 –the day of treatment- and day 5 of treatment; data not shown). The breast and ovarian cancer cell line models studied demonstrated similar sensitivities to these agents.

GLUT1 facilitates basal glucose transport across the plasma membrane. In breast cancer, increased expression of GLUT1 has been reported to be associated with high grade tumors, basal-like subtype, high proliferative index as measured by Ki-67 and poor survival [34–37]. Similarly, an association between high GLUT1 expression and poor outcome has been observed for ovarian cancer [38] suggesting GLUT1 might be a promising target for therapeutic inhibition. The novel GLUT1 inhibitors, STF31 and WZB117 proved inhibitory at lower concentrations when compared to the flavonoids Phloretin and Quercetin. STF31 demonstrated a marked differential effect between the cell lines. It caused a potent cytotoxic effect in the HBL100 breast and TOV112D ovarian cell lines (with an IC_50_ as low as 0.1 μM) whereas the BT549 breast and OVCAR3 ovarian cancer cell lines were found to be highly resistant to the compound. Its activity was first reported in renal cell cancer cell line models [13] and we are unaware of antitumor data being reported in breast or ovarian cancer systems. While the breast cancer cell lines exhibited a similar response to WZB117, with almost identical IC_50_ values, ovarian cancer cell lines showed a significant variation in their response. OVCAR3 cells showed increased sensitivity (IC_50_ 1.4 μM) while OVCAR5 cells were found resistant. In the only published report to date of WZB117, the IC_50_ in MCF-7 cells (approximately 10 μM) was similar to the value (6.4 μM) observed here [14]. (Table 1).

Hexokinase II catalyses the ATP-dependent phosphorylation of glucose. Hexokinase II (HKII), the mitochondrial-bound isozyme, is associated with poor outcome in both breast [39] and ovarian cancers [40]. 3BP proved a potent compound causing a concentration-dependent reduction in the number of viable cancer cells. Breast and ovarian cancer cells presented similar sensitivity. The anti-glycolytic effect of 3BP has previously been demonstrated by decreased FDG uptake in a rat breast cancer model [41]. 3BP has also been shown to have possible value in targeting ovarian cancer tumor initiating cells [42].

PFKFB3 catalyses the ATP-dependent phosphorylation of fructose-6-phosphate and produces Fru-2, 6-BP an allosteric activator of PFK1. It is more highly expressed in breast and ovarian cancers relative to normal tissue [42]. 3PO, a PFKFB3 inhibitor, proved a potent inhibitor in these cell lines. Breast cancer cells lines responded in a similar way while the ovarian cancer cell lines exhibited greater variation in their response. 3PO has already demonstrated promising activity in an MDA-MB-231 breast cancer xenograft model and since these other breast cancer cell lines tested here have increased sensitivity, while the ovarian cancer cell lines have similar sensitivities to the MDA-MB-231 cell line, this would suggest that *in vivo* activity might be obtained in further models [17].

PDHK1 phosphorylates and inactivates PDH and in this way prevents pyruvate from entering the mitochondrial TCA cycle. DCA effectively attenuated cell proliferation in millimolar concentrations in these cell lines and IC_50_ values varied between 5 and 20 mM (Table 1). While it is feasible to achieve concentrations of 1mM in patients [23], these concentrations are insufficient for single agent consideration and combination approaches which require lower concentrations of inhibitors are more likely to be beneficial.

The final target of the glycolytic pathway examined was LDH, a tetramer of A and B subunits that catalyses the reduction of pyruvate to lactate coupled with NADH oxidation. NHI-1 was found to be a much more potent inhibitor than Oxamic acid. Breast and ovarian cancer cells had a similar response to Oxamic acid (Table 1). NHI-1 has been shown to produce synergistic activity when used in combination with gemcitabine in pancreatic cancer cell line models [27] and it may be that combination with cytotoxic drugs would be worth exploring within breast and ovarian cancer models.

The mechanism of action of these inhibitors was also investigated using flow cytometric analysis of treated cells stained with FITC-conjugated Annexin V and Propidium Iodide. Annexin V is a calcium dependent protein that binds to phosphatidylserine when exposed to the extracellular membrane of apoptotic cells. The viability dye PI is excluded from the intact cell membrane of viable cells. With this staining it is possible to distinguish between early apoptotic (Annexin V positive), late apoptotic (Annexin V and PI positive) and necrotic cells (PI positive). Evidence was obtained that all 9 of these inhibitors induce apoptosis. After a 48 h treatment all of them caused an induction of both early and late apoptosis (Figures 5a, 5b), consistent with targeting a common pathway.

The expression levels of the glycolytic targets did not vary markedly in this panel of cell lines (Supplementary Figure 1) and no clear association between expression level and inhibitor response was apparent. We observed though that sensitivity to STF31 (GLUT1 inhibitor) and Oxamic acid (LDHA inhibitor) correlated significantly with the proliferation rate of the cell lines (Figure 6B). The differential effect of these compounds to the various cell lines can be explained by the difference in their growth rate. The most rapidly growing cell lines were found to be more sensitive to these compounds and these are likely to be more dependent on glycolysis for the production of energy and the metabolic intermediates needed for the biosynthesis of macromolecules and for that reason more sensitive to the glycolytic inhibitors.

Seven inhibitors demonstrated a correlation in their IC_50_ values in the panel of cell lines and these were Phloretin, Quercetin, 3BP, 3PO, DCA, NHI-1 and Oxamic acid. This would be consistent with their possessing a common mechanism of action i.e. inhibition of the glycolysis pathway. The two remaining inhibitors, STF31 and WZB117, did not correlate and it is feasible that inhibition of other (non-glycolytic pathway) targets may be responsible for their actions. Certainly, for STF31, a recent report has indicated that it can also inhibit nicotinamide phosphoribosyltransferase (NAMPT) which may contribute to its growth inhibitory activity [43].

Recently, there has been an increasing interest in the anti-proliferative effects of metformin. This widely prescribed antidiabetic drug, because of its low toxicity profile and its established efficacy to target metabolism, has attracted a great deal of attention. A considerable volume of literature has associated metformin with a decreased cancer risk and in addition metformin has been shown in many cases to increase sensitivity to chemotherapy [28, 29]. Currently metformin is undergoing several clinical trials in various cancer types as monotherapy or in combination with other drugs [28]. It has been shown to enhance cisplatin and paclitaxel efficacy in endometrial cancer cells [44] as well as cisplatin efficacy in ovarian cancer cells *in vivo* inhibiting also metastasis in the lung [45]. In another study Iliopoulos *et al*. showed a synergistic effect between metformin and Doxorubicin in a xenograft model using prostate and lung cancer cells [46]. Regarding glycolytic inhibitors two separate research groups, Sahra *et al*. in 2010 and Cheong *et al.* in 2011, demonstrated a synergistic effect between metformin and 2-deoxyglucose (2DG) in prostate cancer cells and mouse xenograft models respectively [47, 48]. Moreover in 2014 Choi *et al*. reported that DCA enhanced metformin potency in inducing cell death in HeLa cells [49].

Encouraged by promising evidence in previous studies we hypothesised that targeting two energy pathways simultaneously; the glycolytic pathway and mitochondrial oxidative phosphorylation, could result in greater energy depletion and enhance cell death of cancer cells. The selected cell line for combination experiments was the triple negative MDA-MB-231. These tumors are considered aggressive and invasive and have limited treatment options [50]. In addition this cell line lacks the functional tumor suppressor gene LKB1 [51]. This is an upstream kinase responsible for the activation of AMPK and is considered important for metformin's antitumor effect [29]. Our results are in agreement with findings from Zordoky *et al*. which showed that MDA-MB-231 cells are sensitive to metformin even though they are LKB1 deficient [51]. In terms of the culture conditions it should be mentioned that these experiments were contacted in 5.56 mM glucose medium equivalent to physiological serum glucose levels, taking into consideration findings from previous studies indicating dependence of metformin's action on glucose availability [49, 51]. We provide evidence of a synergistic interaction between STF31 and metformin as well as between Oxamic acid and metformin (Figure 7A). The CI values generated for both combinations were lower than 0.8 and indicate synergy. The concentrations of 3 and 4 mM Metformin tested are relatively high but millimolar concentrations of the drug have been demonstrated to be achievable *in vivo* as the drug, being stable and not metabolized, can accumulate in tissues at much higher concentrations than in the blood [52]. These findings suggest a potential strategy for triple negative breast cancer treatment.

A key objective of this study was to investigate the sensitivity to the glycolytic inhibitors under varying levels of hypoxia. A published report of a series of normal and malignant breast tissues had indicated a median oxygen level of 8.6% O_2_ in normal breast tissue (*n* = 16) compared to a median value of 4% O_2_ in a series (*n* = 15) of malignant breast cancers [31]. Multiple measurements obtained within individual breast cancers demonstrated values varying between 0 and 13% O_2_ indicating the diversity and mixture of differentially oxygenated populations that co-exist within individual tumors [31]. To test sensitivity, two representative breast cancer cell lines were selected. Dependence on glycolysis was examined over a range of oxygen levels varying from 21% to 0.5% O_2_ and both cell lines examined were more sensitive to all glycolytic inhibitors at 21% O_2_ conditions.

Previous studies investigating these inhibitors in normoxic and hypoxic conditions against other cancer cell lines have demonstrated widely contrasting results with some studies suggesting enhanced activity under normoxic conditions, other studies suggesting enhanced activity under hypoxic conditions and the majority of studies showing equivalent activity or minimally changed activity under both normoxic and hypoxic conditions [53–59]. For example, Phloretin is equally effective under normoxic and hypoxic conditions against the SW620 colon and K562 leukemia cell lines [53] while WZB117 is more effective against hypoxic than normoxic cells [14]. 3BP has previously been shown to be minimally more cytotoxic under hypoxic conditions [54–56] than normoxic conditions although the effects in these studies were very modest and are not dissimilar to other studies which indicate no difference between normoxia and hypoxia [57]. The LDH inhibitor NHI-1 is more effective in hypoxic than normoxic systems [27]. In contrast, several studies have shown that DCA is more cytotoxic in normoxic than hypoxic conditions [58, 59].

Increased dependency under hypoxic conditions might arise through up-regulation of the drug target providing a greater stimulus in cells hence its blockade might be more effective. Equally, increased drug target might also require more inhibitor to block its efficacy and furthermore other pathway components will also be up-regulated by hypoxia, requiring more drug to produce inhibition compared to normoxic conditions. The modulation of the drug targets was examined under these varying oxygen conditions and of the five targets examined, PFKFB3 and PDHK1 were up-regulated under hypoxic conditions in both cancer cell lines, while Hexokinase II and LDHA were up-regulated only in MCDF-7 cells at 0.5% O_2_. Therefore relative resistance to the compounds at low O_2_ levels could be attributed at least in part to increased expression of the targets.

Another factor that should be taken into consideration is that cells grow more slowly in low O_2_ concentration and this might contribute to the decreased sensitivity to the compounds. There were significant associations for most of the compounds between growth rate and drug potency (i.e. IC_50_) across the panel of cell lines (Supplementary Table 3).

Together, these data indicate that inhibition of glycolysis is associated with growth inhibition in these breast and ovarian cancer cell lines. Combination of glycolytic inhibitors with metformin is proposed as a promising strategy for triple negative breast cancer treatment. Variation in oxygen levels between 0.5 and 7% has a relatively small effect on the efficacy of the inhibitors however these compounds are, in general, more effective at 21% O_2_ which appears to correlate with an increased growth rate. We are currently assessing the effects of these inhibitors in combination with various cytotoxic and targeted therapies to assess how these might integrate with established therapies.

## MATERIALS AND METHODS

### Cell culture and inhibitors

A panel of eight cell lines was used; four breast cancer (MCF-7, MDA-MB-231, HBL100 and BT549) and four ovarian cancer cell lines (OVCAR5, TOV112D, OVCAR3, CAOV3). Key features of the cell lines are summarised in Supplementary Table 1. All cell lines were authenticated utilizing Short Tandem Repeat (STR) profiling. They were maintained in Dulbecco's Modified Eagle Medium without HEPES modification (DMEM 31885–023, Invitrogen), including low glucose (5.56 mM), sodium pyruvate (110 mg/L) and L-glutamine (3.97 mM). The medium was supplemented with 10% fetal calf serum and 1% penicillin-streptomycin. The cells were incubated in a humidified incubator at 37°C with 5% CO_2_. They were grown in T175cm^3^ culture flasks, until reaching approximately 70–80% confluence, and were sub-cultured as follows. Medium was removed and cells were washed with phosphate buffered saline (PBS). Trypsin was used to cause cell detachment. Medium containing serum was added to neutralize the enzyme and cells were pelleted at 400G for 5min. Finally, cells were resuspended in media and passaged into fresh flasks or dishes (ratio 1:10). When necessary, cells were counted using a Neubauer hemocytometer. All procedures were performed under sterile conditions in a Laminar Air Flow hood. Hypoxia experiments were conducted at 37°C with 5% CO_2_ and 7%, 2% or 0.5% O_2_ using the H35 Hypoxystation (Don Whitley Scientific, Shipley, UK). Prior to hypoxia experiments, cells were allowed to adapt to each oxygen level for at least 5 days.

Phloretin, Quercetin, 3BP, DCA and Oxamic acid were obtained from Sigma Aldrich. STF31 and Metformin were obtained from Tocris Bioscience, WZB117 and 3PO from Merck Millipore and NHI-1 from Mercachem. Stock solutions of compounds were prepared in DMSO except for WZB117 which was dissolved in ethanol and DCA, Oxamic acid, 3BP and Metformin in PBS.

### Sulphorhodamine B assay (SRB)

The SRB assay is a cell density assay based on the measurement of cellular protein content [60]. Cells (0.5 – 2 × 10^3^ cells/well, depending on the proliferation rate of each cell line) were seeded in 96-well plates. Forty-eight hours later, cells were treated with or without the indicated concentration of the inhibitors. A 10-point dilution series with 1:2 steps in six replicates was performed. For the compounds dissolved in dimethylsulphoxide (DMSO) or ethanol, a constant DMSO/ethanol concentration was used across the whole concentration- response curve. After a five day incubation period, cell monolayers were fixed with cold 25% trichloroacetic acid (Sigma) and stained with the SRB dye solution (Sigma). Unbound excess dye was removed by 1% acetic acid washes. The protein-bound stain was solubilized in 10mM Tris buffer solution (pH 10.5). Finally absorbance was measured at 540 nm using a microplate reader (BP800, Biohit Health Care). Measurements were corrected for background absorbance and presented as percentage of absorbance in untreated cells

The half maximal inhibitory concentration (IC_50_) was used as a measure of the effectiveness of each compound. It indicates the concentration needed to reduce cell number by half. Sigmoidal concentration response curves were fitted and the IC_50_ values were defined using the XL fit tool within the Microsoft Excel.

For drug interaction studies a range of 7 different concentrations of glycolytic inhibitors was used in combination with a fixed concentration of metformin (around the IC_20_). Both drugs were administered at the same time and the SRB assay was performed after a three day treatment. Data were analysed using the Calcusyn Software generating combination index values (CI) for each single combination point [61].

### Glucose uptake assay

Cells were seeded in 12-well plates at 1 × 10^5^ to 2 × 10^5^ cells per well. The following day, cells were treated with or without the indicated concentration of inhibitor. The concentrations used for each compound were determined based on the corresponding IC_50_ values derived from the SRB assays. Culture media was collected at 24 h. Glucose remaining in the media was measured using the Amplex Red Glucose/Glucose Oxidase Assay Kit (Invitrogen), according to the manufacturer's instructions. Samples were diluted 1:50 in 1xReaction Buffer provided in the kit. A 96-well plate format with triplicates was used. After a 30 min incubation period with the reaction reagent solution, protected from light, absorbance was measured at 540 nm using the microplate reader. Measurements were corrected for background absorbance, subtracting the value derived from the no-glucose control [62].

### Lactate assay

Lactate was measured in the same samples as used for the glucose assay, as described above. Lactate produced in the media was measured using the Lactate Assay Kit (Trinity Biotech), according to the manufacturer's instructions. A 96-well plate format with triplicates was used. 2 μl of sample was added to the wells followed by 200 μl of lactate reagent. The reagent was used as a 50% solution in distilled water. Plates were incubated in the dark for 7 min and absorbance was measured at 540 nm using the microplate reader. Measurements were corrected for background absorbance, subtracting the value derived from the no-lactate control [63].

### Flow cytometry analysis

Detection of compound-induced cell death was carried out by dual staining with FITC-conjugated Annexin V and Propidium Iodide (PI) followed by flow cytometry. Briefly, 3 × 10^5^ cells were seeded in 6-well plates and the following day they were treated with or without the indicated concentration of inhibitor. After a 48 h treatment, cells were harvested and stained using the FITC Annexin V Apoptosis Detection Kit I (BD Pharmingen) according to the manufacturer's instructions. Data acquisition and analysis of 30,000 events for each sample was performed using the flow cytometer BD Accuri C6 (BD Biosciences). Annexin V single positive cells were identified as early apoptotic, while cells both Annexin V and PI positive were identified as end stage apoptotic cells and PI positive cells as necrotic.

### Western blotting

Cells were seeded in cell culture dishes and, when approximately 80% confluent, were washed with ice cold PBS and then treated with ice cold isotonic lysis buffer (50mM Tris pH7.5, 5 mM EGTA pH8.5, 150 mM NaCl, 1 “Complete Protease Inhibitor Tablet” (Roche), 100 μl Phosphatase Inhibitor Cocktail 2 (Sigma), 100μl Phosphatase Inhibitor Cocktail 3 (Sigma), 50 μl Aprotinin (Sigma), 100 μl Triton-X 100 (Sigma)). Subsequently cells were scraped and after centrifugation lysate supernatant was collected and stored at −70°C. The Bicinchoninic Acid Assay (BCA) was performed to determine lysate protein concentration and absorbance was measured at 540nm using a microplate reader. Sodium dodecyl sulphate polyacrylamide gel electrophoresis (SDS-PAGE) was performed to separate proteins according to their molecular weight. Following electrophoresis, proteins were transferred onto a methanol-activated Immobilon-P polyvinylidene fluoride (PVDF) Transfer Membrane (Immobilon) using the Bio-Rad Protean Transfer Cell equipment. Transfer was performed at 100V for 1 h in ice-cold Tris-Glycine transfer buffer at 4°C. After transfer the membrane was blocked for 1 h at RT, in 1:1 PBS/Odyssey Blocking Buffer to prevent non-specific binding. Following blocking, the membrane was incubated in primary antibody solution at 4°C overnight. The following primary antibodies were used: GLUT1 (07-1401; Millipore), HKII (2867; CST), PFKFB3 (13123; CST), PDHK1 (3820; CST), LDHA (3582; CST) and α-Tubulin (ab7291; Abcam). As antibody diluent, PBS/Odyssey Blocking Buffer or 5%w/v bovine serum albumin (BSA, Sigma) in 1X PBS containing 0.1% Tween-20 were used. After primary antibody incubation, the membrane was washed with PBS-0.1% Tween 20, to remove excess antibody. Secondary fluorescent antibody (anti-rabbit IR Dye 800CW (926-32211; Odyssey) and anti-mouse IR Dye 680LT (926-68020; Odyssey)) were employed, raised against the species of the primary antibody and diluted in PBS/Odyssey Blocking Buffer containing 0.001% SDS. Incubation was performed for 45 min at room temperature, protected from light. Finally the membrane was washed with PBS-0.1% Tween 20. Visualization of proteins was achieved by scanning on the Odyssey Infrared Imaging System (Licor). This machine is equipped with two infrared channels for direct fluorescence detection allowing two separate targets to be probed simultaneously.

### Statistics

To evaluate the significance of differences between treated samples and untreated controls, ANOVA followed by the Tukey-Kramer Multiple Comparisons Test was used. To correlate the expression of the glycolytic enzymes in the panel of the eight cancer cell lines with their sensitivity to the inhibitors the non-Parametric Spearman correlation test was performed. Statistical tests were undertaken using GraphPad software.

## SUPPLEMENTARY FIGURES AND TABLES


