# Supplementary material for: A comparative analysis of inhibitors of the glycolysis pathway in breast and ovarian cancer cell line models
Source: Oncotarget. 2015 Jul 16;6(28):25677–95. doi: 10.18632/oncotarget.4499 (PMC4694858; doi:10.18632/oncotarget.4499)
Supplement: Supplementary file 1 [file oncotarget-06-25677-s001.pdf]

SUPPLEMENTARY FIGURES AND TABLES

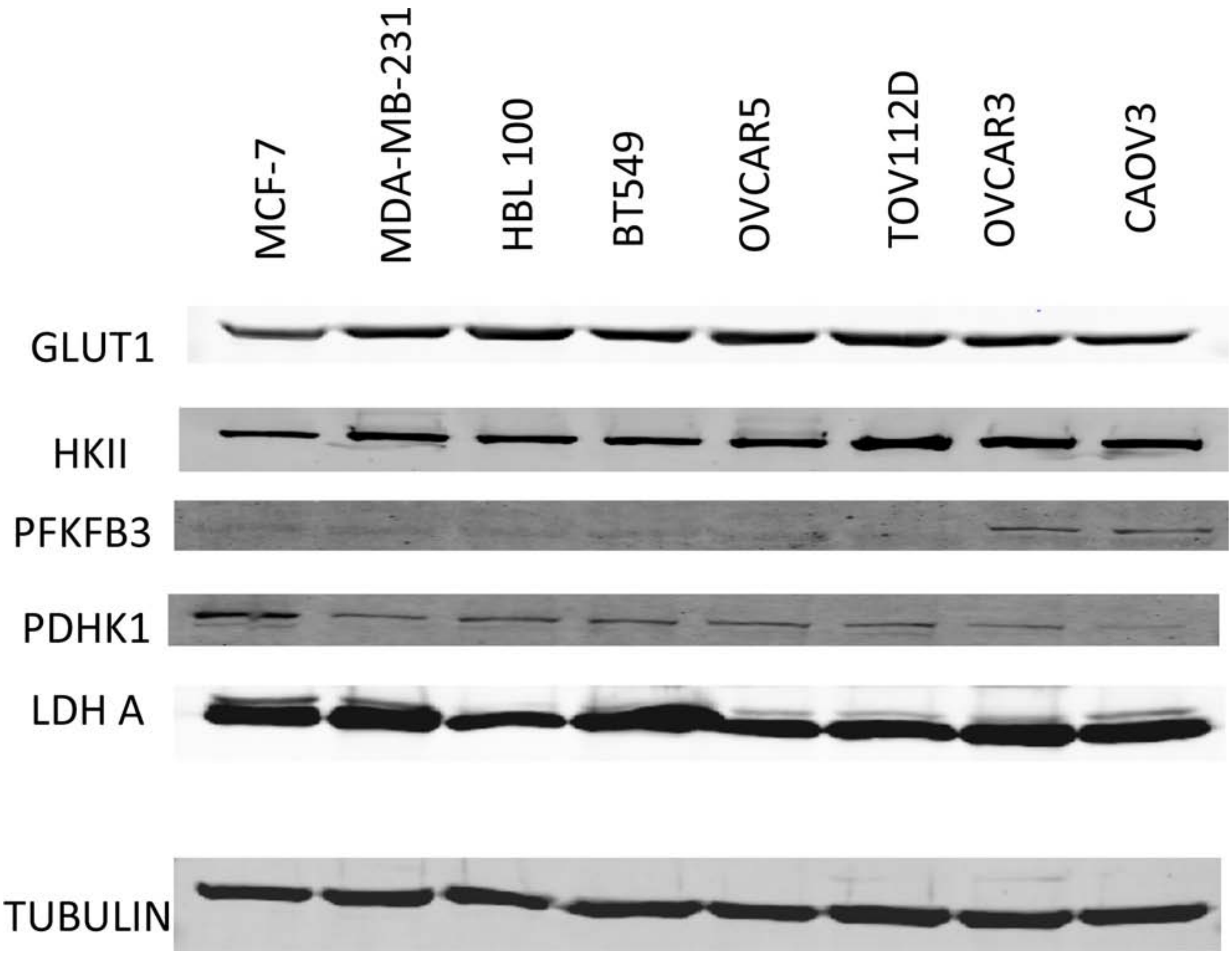

Supplementary Figure S1: GLUT1, HKII, PFKFB3, PDHK1 and LDHA expression were examined in the eight cell lines. Tubulin expression was examined in the same samples as a loading control.

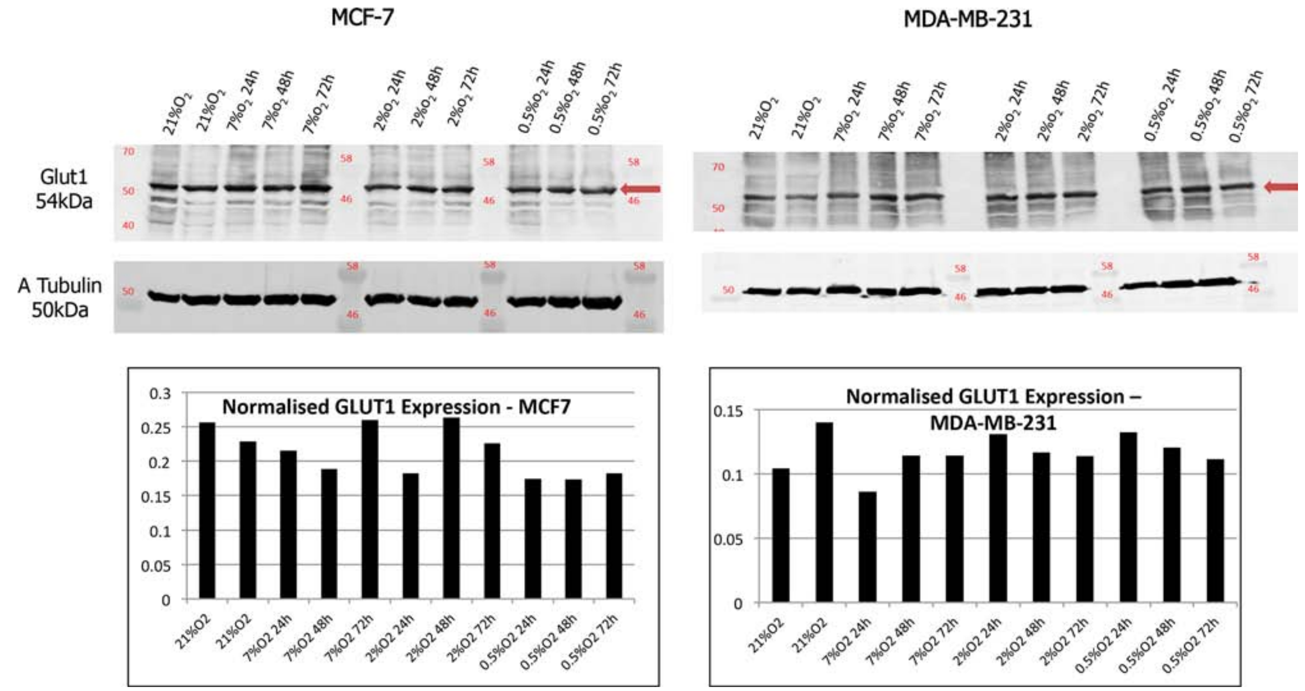

Supplementary Figure S2: GLUT1 expression was examined in MCF-7 and MDA-MB-231 cells at different O_2 levels. Lysates were taken from cells cultured in 0.5% O_2, 2% O_2 and 7% O_2 for different periods of time, 24 h, 48 h and 72 h. Samples are presented as follows: 21% O_2, 21% O_2, 7% O_2 24 h, 7% O_2 48 h, 7% O_2 72 h, 2% O_2 24 h, 2% O_2 48 h, 2% O_2 72 h, 0.5% O_2 24 h, 0.5% O_2 48 h and 0.5% O_2 72 h. Tubulin expression was examined in the same samples as a loading control. Densitometric analysis of GLUT1 expression was performed using the Odyssey Infrared Imaging System software (Licor).

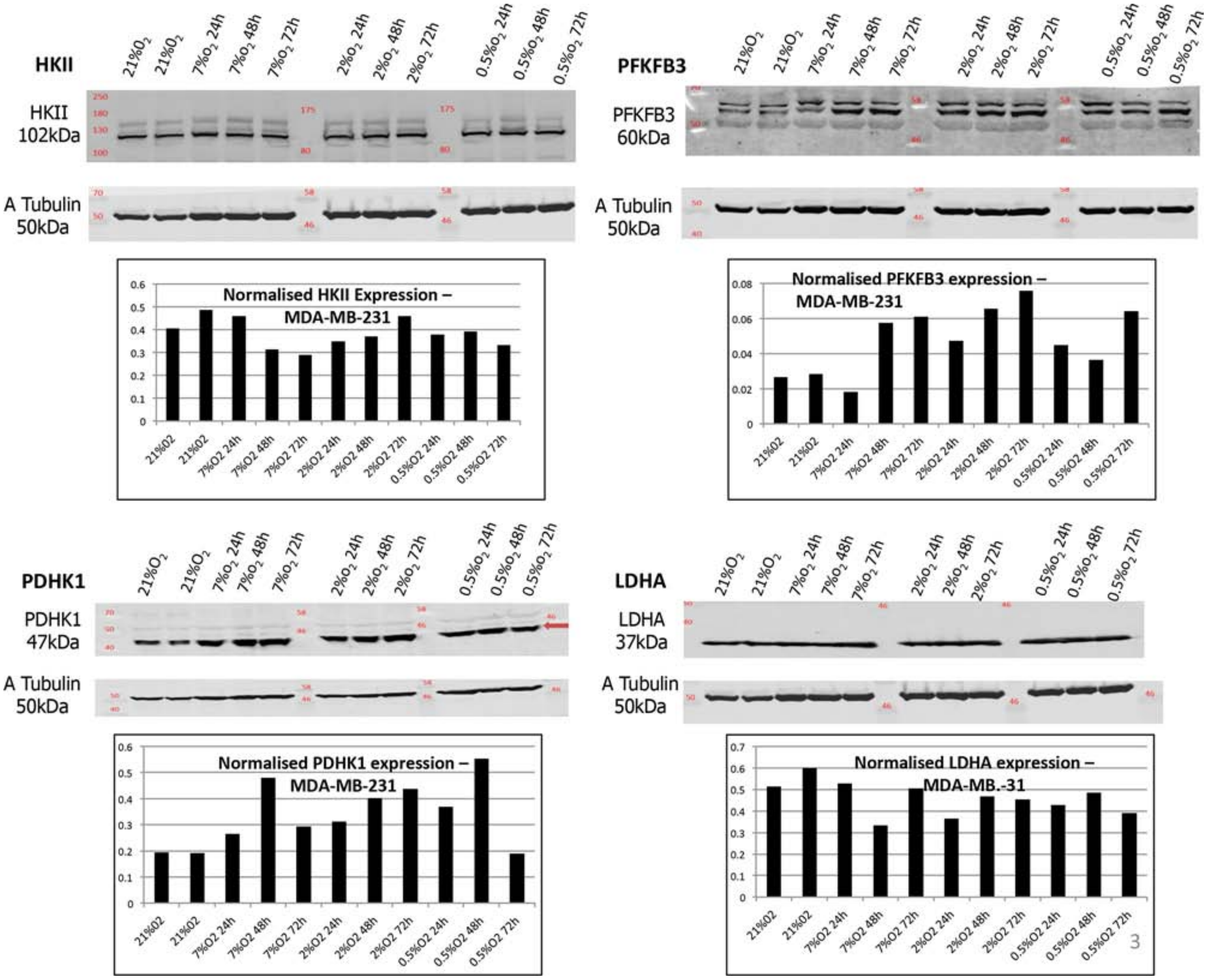

Supplementary Figure S3: HKII, PFKFB3, PDHK1 and LDHA expression were examined in MDA-MB-231 cells at different O_2 levels. Lysates were taken from cells cultured in 0.5% O_2, 2% O_2 and 7% O_2 for different periods of time, 24 h, 48 h and 72 h. Samples are presented as follows: 21% O_2, 21% O_2, 7% O_2 24 h, 7% O_2 48 h, 7% O_2 72 h, 2%O_2 24 h, 2% O_2 48 h, 2% O_2 72 h, 0.5% O_2 24 h, 0.5% O_2 48 h and 0.5% O_2 72 h. Tubulin expression was examined in the same samples as a loading control. Densitometric analysis of HKII, PFKFB3, PDHK1 and LDHA expression was performed using the Odyssey Infrared Imaging System software (Licor).

Supplementary Table S1. Characteristics of the breast and ovarian cancer cell lines

Cell Line	Cancer Subtype	Receptor Status*
MCF-7	Breast Luminal	ER^+, PR^+
MDA-MB-231	Breast Basal B	ER^-, PR^-, $HER2^-$
HBL100	Breast Basal B	ER^-, PR^-
BT549	Breast Basal B	ER^-, PR^-
OVCAR5	Ovarian	
TOV112D	Ovarian endometrioid	
OVCAR3	Ovarian	
CA0V3	Ovarian	

*(adapted from Neve *et al*., 2006).

Supplementary Table S2. Correlation between IC_{50} concentrations for pairs of drugs tested against the panel of cell lines (Pearson *p*-values shown)

	Phloretin	Quercetin	STF31	WZB117	3-BP	3-PO	DCA	Oxamic acid	NHI-1
**Phloretin**		**0.018**[*]	0.53	0.93	**0.017**	**0.010**	0.22	**0.049**	0.19
**Quercetin**	**0.018**		0.88	0.94	0.07	**0.015**	0.14	0.097	**0.019**
**STF31**	0.53	0.88		0.63	0.62	0.88	0.89	0.71	0.99
**WZB117**	0.93	0.94	0.63		0.99	0.58	0.38	0.98	0.38
**3-BP**	**0.017**	0.07	0.62	0.99		**0.024**	0.24	**0.002**	0.29
**3-PO**	**0.010**	**0.015**	0.88	0.58	**0.024**		**0.021**	**0.009**	**0.03**
**DCA**	0.22	0.14	0.89	0.38	0.24	**0.021**		0.21	**0.011**
**Oxamic acid**	**0.049**	0.097	0.71	0.98	**0.002**	**0.009**	0.21		0.30
**NHI-1**	0.19	**0.019**	0.99	0.38	0.29	**0.03**	**0.011**	0.30	

[*]Statistically significant values ($p < 0.05$) shown in bold

Supplementary Table S3. Summary of the IC_{50} concentrations presented from two breast cancer cell lines when treated with the indicated glycolytic inhibitors for 5 days under four different oxygen conditions (21% O_2, 7% O_2, 2% O_2, and 0.5% O_2) and association with the growth rate in the respective conditions (Pearson *p*-values shown)

IC_{50}	MCF-7				MDA-MB-231				Pearson *P* value*
	21% O_2	7% O_2	2% O_2	0.5% O_2	21% O_2	7% O_2	2% O_2	0.5% O_2	
**STF31 (µM)**	1.3	2.3	1.4	1.6	1.2	1.8	2.4	2.2	0.0304
**WZB117 (µM)**	2.3	16	>30	>30	4.1	N/A	>30	N/A	0.0014
**Phloretin (µM)**	90	295	>300	>300	70	289	>300	>300	0.0030
**3 BP (µM)**	63	>300	N/A	>300	39	>300	N/A	>300	0.0031
**3PO (µM)**	1.3	3.8	3.5	3.7	1.5	11	8.6	13	0.0135
**DCA (mM)**	13	19	20	13	23	39	37	31	0.0550
**NHI-1 (µM)**	62	80	150	111	94	190	228	174	0.0037
**Oxamic acid (mM)**	10	39	47	49	6.9	60	66	74	<0.0001
**Control growth rate****	16.3x	10.7x	9.0x	10.7x	15.7x	7.9x	5.6x	6.8x	
